# Face mask use as a categorical dimension in social perception

**DOI:** 10.1038/s41598-022-22772-2

**Published:** 2022-10-25

**Authors:** Luigi Castelli, Matilde Tumino, Luciana Carraro

**Affiliations:** Department of Developmental and Social Psychology, University of Padova, Via Venezia 8, 35131 Padua, Italy

**Keywords:** Psychology, Human behaviour

## Abstract

Prevention measures aimed at combating COVID-19 pandemic strongly impact several aspects of social life. In particular, interpersonal perception is affected as a function of whether the persons perceived wear or not face masks. In two experimental studies, we here explored whether people rely on the presence vs. absence of face masks when encoding information in memory about other individuals. In a memory confusion paradigm, participants were initially presented with individuals either wearing a face mask or not, each conveying a series of sentences. Next, participants were probed about the identity of the speaker of each sentence. Results showed that it was more likely to erroneously attribute a sentence to a speaker who also was wearing a face mask (or not) as the original speaker, demonstrating that the cue about wearing or not a face mask was spontaneously used to encode information. Study 2 ruled out an alternative explanation based on perceptual processes, suggesting that face masks represent meaningful social objects. Overall, it emerged that participants spontaneously categorize others as a function of whether they wear a mask or not. Findings also confirmed previous research evidence about the more positive evaluation of mask wearers as compared to non-wearers, and the overall detrimental impact that face masks may have on the correct identification of social targets.

## Introduction

The sudden outbreak of the COVID-19 pandemic has forced countries all around the world to adopt unprecedented measures in order to limit the spread of the virus and protect public health^1^. This global challenge has required the introduction of a host of drastic changes in people’s lives, such as restrictions in social contacts, an increased attention to interpersonal distance, the use face masks, and the need to decide to either join or not vaccination campaigns. Research has widely investigated both the psychological determinants of people’s propensity to comply with these behavioral requirements^2–6^, and their effects on psychological well-being^7,8^ as well as on socio-cognitive processes^9,10^. A specific attention has been devoted to the impact that the use of protective face masks may have on several key aspects of cognition and social life^9^. At the level of interpersonal perception, emotion recognition appears to be severely impaired due to the reduced relevant visual information that can be extracted from faces wearing masks^11,12^. Although the overall recognition of basic emotional expressions remains above chance level even in the case of mask wearers, it tends to be considerably hindered for most emotions^13–15^. In a similar vein, it has been suggested that face masks interfere with identity recognition, similarly to other manipulations that obscure relevant parts of the face^16^. Indeed, it has been shown that identity recognition can be significantly reduced when both familiar and unfamiliar targets wear face masks^17–23^. For instance, Carragher and Hancock^18^ have shown that people are far less accurate to determine whether two simultaneously presented face photographs portray the same person or two different people when at least one of the two targets is wearing a face mask. Hence, an accurate interpersonal perception can be impoverished due to the presence of face masks.

Social judgment after the emergence of Covid-19 pandemic appears to be also shaped by the presence vs. absence of face masks on the evaluation targets. Indeed, individuals wearing a face mask tend to be judged as more attractive, approachable, and trustworthy^14,24–28^, although some studies failed to report similar effects^17,29–31^. The positive evaluation of mask wearers found in previous research is likely to reflect the fact that mask wearing is seen as a normative behavior^32^. The perceived normativity of mask wearing is indeed associated with overall pro-mask attitudes^33,34^, and the more people use protective facial masks in their everyday life, the more positive their attitudes towards facial masks tend to progressively become^35^. In addition, behaviors aimed at combating the dramatic consequences of COVID-19 have become moralized^36^, thus affecting the moral evaluation of people who either comply or deviate from the prescribed normative behaviors^37^. This, of course, does not necessarily imply that there is a ubiquitous consensus about the importance of mask use^38^. For instance, there is evidence that the attitudes towards the use of face masks are strongly politicized and more conservative individuals typically hold more negative views in relation to the use of these protective gears^33,39^.

### The goals of the present research

Previous research has outlined the impact of presence of masks on face and emotion processing, as well as on deliberate trait inferences. So far, however, little is known about whether people spontaneously make use of this dimension while processing and encoding social information in memory. The major aim of the present work is therefore to assess whether people organize information in memory as a function of the mask-related behaviors of the person perceived. To this end, we employed a specific memory confusion paradigm—often called “who-said-what” task—which has been widely applied to investigate social categorization processes^40–43^. In the typical task, participants are required to attend to a sequence of speakers each expressing a predefined number of sentences, and afterwards to try to match each sentence with the actual original speaker. Critically, speakers differ from each other along one or more categorical dimensions (e.g., race or gender). The main dependent measure of interest is related to the nature of the memory confusions that can take the form of either within-category errors (i.e., a sentence is erroneously attributed to an individual belonging to the same category as the original speaker) or between-category errors (i.e., a sentence is erroneously attributed to an individual belonging to a category the original speaker is not member of). Following the logic of the paradigm proposed by Taylor et al.^43^, we here speculated that if people do indeed spontaneously categorize speakers as either mask wearers or non-wearers, we should expect within-category errors to be more likely than between-category errors. For instance, when correct recognition fails, a statement initially provided by a mask wearer should be more likely attributed to another individual wearing the mask (i.e., within-category error) than to an individual who does not wear the mask (i.e., between-category errors). In other words, if participants initially encoded information in memory with a tag related to the presence vs. absence of a mask on the speaker’s face, this should later increase within-category errors. Beyond this general effect, in Study 1 we also aimed to explore whether the hypothesized organization of information in memory as a function of the presence (or not) of a mask on the speakers’ face is modulated by making the issue of face masks salient or not. To this end, half of the participants were asked to think and report their attitudes towards the use of face masks before performing the who-said-what task. The other half of the participants went directly to the who-said-what task, and only afterward reported their attitudes. This will allow to ascertain whether people spontaneously use the cue about the presence vs. absence of the face mask also when they are not prompted to preliminary focus on such issue.

Although the primary focus of the present research was on the spontaneous use of the presence vs. absence of face masks when encoding information in memory, the employed memory task also allows to examine the pattern of correct responses. As discussed above, the presence of face masks can heavily impair identity recognition^18^. It is here hypothesized that source monitoring could also be affected, so that the ability to correctly match a statement with the actual identity of the original speaker would be weakened by the presence of face masks.

Finally, because some previous evidence points to the presence of more positive evaluations towards targets wearing face masks as compared to targets who do not wear them^14,24–28^ whereas other studies reported diverging findings^17,29–31^, in Study 1 we aimed at further exploring the effects of face masks on explicit evaluations. Given the inconsistency between previously published findings, however, we did not formulate specific hypotheses. In addition, because the issue of preventive behaviors against Covid-19 has often been politicized in several countries including Italy^32,33,38,44,45^—with conservative individuals being more reluctant to adopt such behaviors—we also assessed participants’ political orientation. In line with previous research, we expected that more conservative respondents would express more negative attitudes towards the use of face masks in general and towards mask wearers (vs. non-wearers).

## Study 1

### Participants

A power analysis indicated that 171 participants would be required to detect a relatively small effect (*d* = 0.25) in relation to the comparison between within- and between-category errors, with alpha set at 0.05 and power = 0.90. We decided to recruit 200 participants in order to account for exclusions. Participants (96 females, 101 males, 3 other) had a mean age of 27.04 years (*SD* = 8.20), ranging from 18 to 63 years. The experiment was run on Qualtrics and the data were collected through the crowdsourcing platform Prolific. All participants were Italian native speakers and they all provided informed consent before starting the experiment. The study was approved by the Psychology Ethic Committee at the University of Padova. All methods were carried out in accordance with relevant guidelines and regulations. Data, materials, and additional information about the sample are available at https://osf.io/8u2yz/?view_only=da3d7536ed114101bbb76bb85f766856. The study was run in December 2021.

### Measures

#### Memory confusion task

**Figure 1:**
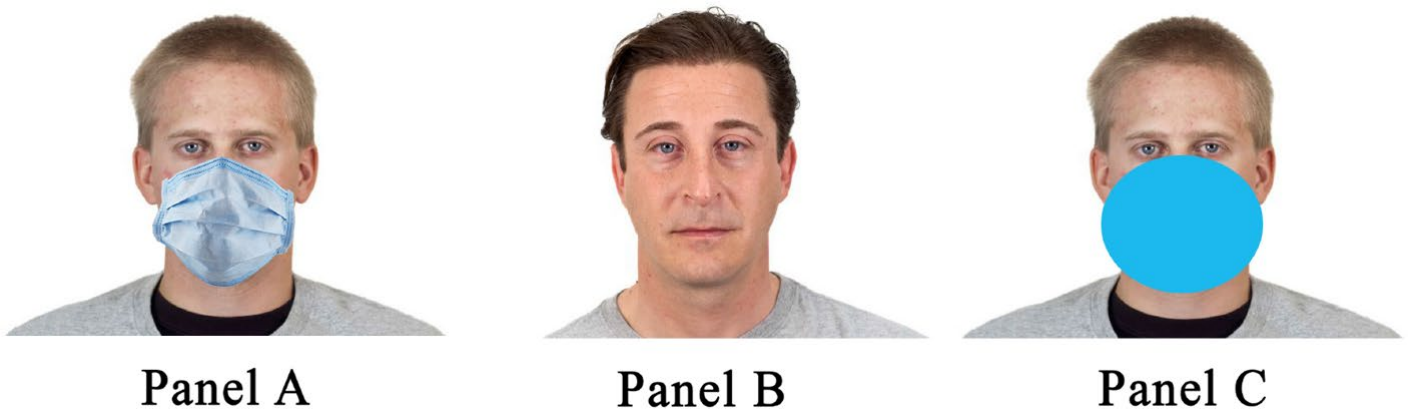
Examples of the face stimuli used in Study 1 (panel **A** and **B**) and in Study 2 (panel **A**, **B**, and **C**). Stimuli without the mask/spot were extracted from the Chicago Face Database^46^—complying with the terms of use—and then edited to superimpose the face mask or the spot.

Participants were initially required to go through a presentation phase in which they were shown a sequence of 24 sentences allegedly pronounced by 8 different White male speakers. Participants were presented with the image of the speaker appearing at the center of the screen and the statement was written right below the image. The speaker’s photograph and the sentence remained visible for 7 s. Importantly, 4 speakers wore a surgical face mask whereas 4 speakers had no face mask (see Fig. 1, panel A and B). The pictures of the speakers were retrieved from the Chicago Face Database^46^ and all had a neutral expression (see Materials at https://osf.io/8u2yz/?view_only=da3d7536ed114101bbb76bb85f766856). Whether a specific speaker wore the face mask or not was counterbalanced across participants. Face masks were added using Adobe Photoshop. The order of presentation of the speaker-sentence pairings was randomized. Participants were instructed to carefully attend the presentation because they would be later asked to perform a memory task. The sentences referred to neutral common behaviors (e.g., “I had breakfast with a cup of milk and a croissant”).

After this presentation, participants were presented with each of the 24 sentence—one at the time in random order—and required to select the picture of the speaker who actually expressed it. The pictures of the 8 speakers displayed during the presentation phase, were all shown on the screen next to each other in a 2 (horizontal) × 4 (vertical axis) matrix. The display with the 8 speakers was centered on the screen and the specific location of each speaker within the 2 × 4 matrix randomly changed on each trial so that to encourage careful inspection. Accuracy was stressed and there was no time constraint while performing the task.

#### Attitudes towards face mask use

Participants were asked to fill in a 9-items scale aimed at assessing the general appraisal of individuals who wear a surgical face mask (e.g., *“When I see a person who does not wear a face mask, I immediately think that he/she is selfish”*; *“Using the face mask is a sign of respect for others”*). Responses were provided by indicating the level of agreement/disagreement with each sentence along a continuum ranging from 0 (= totally disagree) to 100 (= totally agree). Reliability was good (α = 0.89) and a summary score was computed (*M* = 68.27, *SD* = 19.72).

#### Explicit evaluation of the speakers

The same 8 speakers presented in the who-said-what task were shown again in a random order, and for each of them participants were required to report the perceived trustworthiness, morality, sociability, competence, and altruism. Responses were provided along a continuum ranging from 0 (= not at all) to 100 (= very much).

#### Political orientation

Participants were asked to report their global political orientation on a continuum from 0 (= left-wing) to 100 (= right-wing). The same continuum was used in two additional questions aimed at assessing participants’ political orientation in relation to social and economic issues, respectively. Responses to the three questions were highly correlated and a summary score was computed (α = 0.87; *M* = 31.58, *SD* = 20.24).

### Procedure

Half of the participants performed the memory confusion task before the assessment of the attitudes towards the use of face masks and the evaluation of the speakers. The other half of the participants started filling in the scale about the attitudes towards the use of face masks before moving to the memory confusion task and the evaluation of the speakers. The assessment of political orientation and demographic questions were always placed at the end.

Ethics and consent: Informed consent was obtained from all participants. The research was approved by the Psychology Ethic Committee of the University of Padova (Protocol #4113). All methods were carried out in accordance with relevant guidelines and regulation.

### Results

All the reported analyses were two-tailed and the alpha level was set at 0.05.

#### Memory confusion task

The data from 7 participants were excluded from the analyses because they provided random responses in the memory task (i.e., 3 or less correct answers) indicating that they did not pay adequate attention during the presentation phase. This left 97 participants in the condition in which the attitudes towards the use of face masks were assessed before the memory task and 96 participants for whom the measures were administered in the reverse order.

We first focused on errors. As in the standard memory confusion protocol^43^ we calculated the number of within-category and between-category errors. A within-category error occurred when the original speaker wore the mask and the sentence was erroneously attributed to another speaker with the mask, or when both the original speaker and the speaker to whom the sentence was erroneously attributed did not wear the mask. Between-category errors, in contrast, occurred when the original speaker and the speaker to whom the sentence was erroneously attributed differed in relation to the presence vs. absence of the face mask. In order to correct for baseline probabilities, in line with Taylor et al.^43^, between-category errors were multiplied by 3/4. Indeed, only 3 targets could be associated to a within-category error being the fourth target the correct answer.

These two indexes were submitted to a 2 (type of error: within- vs. between-category) × 2 (condition: scale about face mask use administered before vs. after the memory confusion task) analysys of variance with the first factor varying within participants and the second between participants. A main effect of the type of error emerged, *F*_(1, 191)_ = 16.33, *p* < 0.001, *η*^2^_*p*_ = 0.079. Indeed, within-category errors (*M* = 6.13, *SE* = 0.19) were significantly more likely than between-category errors (*M* = 5.17, *SE* = 0.16). Neither the main effect of condition, *F*_(1, 191)_ = 1.46, *p* = 0.228, *η*^2^_*p*_ = 0.008, nor the interaction effect, *F*_(1, 191)_ = 0.017, *p* = 0.895, *η*^2^_*p*_ = 0.000 were significant. This strongly suggests that participants encoded information as a function of the presence vs. absence of the face mask to the same extent in both conditions. Because there was relevant interindividual variability in the overall number of errors—thus making the responses of some participants potentially more impactful in the analyses—we also analyzed the data following a different strategy. More specifically, the proportion of within-category errors out of the overall number of errors was separately calculated for each participant. The observed value was then compared to the expected value in case responses would have been unaffected by the presence vs. absence of the face mask (i.e., 3/7 = 0.4285). A one-sample t-test confirmed that within-category errors (*M* = 0.4717, *SD* = 0.16) were indeed more likely than chance, *t*_(191)_ = 3.84, *p* < 0.001, *d* = 0.28.

Next, we analyzed correct responses with the goal to ascertain whether the correct pairing between a speaker and the sentence he had conveyed was somehow impaired when the speaker was wearing a face mask. To this end, we carried out a 2 (speaker: with vs. without the mask) × 2 (condition: scale about face mask use administered before vs. after the memory confusion task) analysys of variance on correct responses, with the first factor varying within participants and the second between participants. The presence vs. absence of the mask yielded a significant main effect, *F*_(1, 191)_ = 16.327, *p* < 0.001, *η*^2^_*p*_ = 0.079. Indeed, correct responses were more likely when the original speaker had no face mask (*M* = 5.80, *SE* = 0.17) as compared to when a face mask was present (*M* = 5.17, *SE* = 0.17). No other effect was significant (*p*s > 0.23).

#### Explicit evaluation of the speakers

Data were submitted to a 2 (face mask: present vs. absent) × 5 (trait: trustworthiness, morality, sociability, competence, and altruism) analysis of variance with all factors manipulated within participants. A significant main effect of face mask emerged, *F*_(1, 191)_ = 140.37, *p* < 0.001, *η*^2^_*p*_ = 0.424, indicating more positive evaluations towards targets using the mask. The effect of the trait, *F*_(4, 188)_ = 4.97, *p* < 0.001, *η*^2^_*p*_ = 0.096, and the interaction effect between face mask and trait, *F*_(4, 188)_ = 11.21, *p* < 0.001, *η*^2^_*p*_ = 0.193, were also significant. Separate analyses as a function of the specific trait showed that the preference for mask wearers was significant for all dimensions (4.89 < *t*s < 11.63; *p*s < 0.001), although it was slightly weaker for sociability (*d* = 0.35) as compared to the other traits (0.72 < *d*s < 0.84).

#### Relationship between the measures

Correlation analyses have been performed in order to explore the relationship between the index based on the proportion of within-category errors and the attitude towards face mask use, the political orientation and the overall explicit evaluation of the speakers wearing the mask (α = 0.90) or not wearing it (α = 0.91). No significant correlation emerged (see Table 1). The composite index about political orientation was negatively correlated with the attitude towards face mask use, *r*_(200)_ = -0.22, *p* = 0.002 (i.e., conservatives reported less positive attitudes). A regression analyses in which the responses to the economic and social conservatism items were simultaneously entered as predictors showed a significant effect of the economic conservatism, *β* = -0.172, *p* = 0.035, 95% CI [-279, -0.01], whereas social conservatism was not a significant predictor of the attitude towards face mask use (*p* = 0.35). In no case political orientation correlated with the explicit evaluation of the speakers with either the mask or not. The responses in the scale aimed at assessing the attitudes towards face mask use were positively correlated with the evaluation of mask wearers, *r*_(199)_ = 0.48, *p* < 0.001, but they were substantially unrelated to the perception of targets who did not wear the mask, *r*_(199)_ = 0.057, *ns*.

**Table 1:** Correlations in Study 1.

	1	2	3	4	5
1. Proportion of within-category errors	–				
2. Attitudes towards face mask use	−.049	–			
3. Explicit evaluation of mask wearers	.025	.478**	–		
4. Explicit evaluation of mask non-wearers	−.080	.057	.336**	–	
5. Political orientation	.061	−.218*	−.114	−.010	–

*p < .005; **p < .001.

### Discussion

Results strongly supported the hypothesis that participants encoded information in memory with a tag related to the presence vs. absence of a mask on the speaker’s face. It thus appears that there was a spontaneous use of this information as an organizing principle in memory, and the effect was not influenced by a manipulation aimed at focusing participants’ attention on the issue of face masks.

In addition, findings further confirmed that the presence of face masks disrupts memory processes that involve the identification of social actors. In particular, participants were here far less likely to correctly identify the speaker conveying a specific sentence when he was wearing a face mask. This suggests that source monitoring processes can thus be impaired when social actors are wearing a face mask. Despite this negative outcome associated to the use of face masks, mask wearers were pervasively evaluated more positively as compared to individuals not wearing the mask^27,28^.

Although findings from this study indicated that face masks were used as a cue to encode information in memory, it remains unclear whether this effect reflected a meaningful social process. We argued that participants spontaneously processed targets in terms of the adoption—or not—of a relevant social behavior (i.e., the use of face masks), and, as an outcome of this process, they tagged the received information accordingly. However, the study could not rule out an alternative explanation based on the mere perceptual difference between the two sets of pictures (i.e., the faces with the mask and those without it). For instance, two images of speakers wearing the mask are perceptually more similar to each other as compared to the image of a speaker wearing a mask and one who does not wear it, because both images display the same colored object (i.e., the light blue mask). Importantly, Stangor et al.^42^ employed a memory confusion paradigm and demonstrated that a meaningless perceptual dimension (i.e., the color of the clothing) was not used to categorize targets when another relevant social dimension was present (i.e., race; see also^47^). In the current study, however, the presence vs. absence of the mask was the only available cue for categorization and it also involved the face of the targets, namely the physical area within the picture that participants were likely to more carefully inspect in order to encode the identity of the speaker. Therefore, as a means to tease apart the “social” and the “perceptual” explanations and provide a stringent test of our hypothesis, in Study 2 we introduced two different conditions that were manipulated between participants. One condition basically replicated Study 1, with half of the presented faces covered by a face mask and the other half without face masks. In the other condition, faces did not wear face masks, but half of them had their lower part covered by a large spot of about the same size and color of the masks that were employed in the other condition. In this way, in both conditions half of the faces had the same area occluded by a superimposed stimulus, but only in one condition it was a meaningful social object (i.e., the protective face mask).

## Study 2

### Participants

Two-hundred participants (82 females, 115 males, 3 other; age = 27.33 years, *SD* = 7.80, from 18 to 65 years) were recruited. The experiment was run on Qualtrics and the data were collected through Prolific. All participants were Italian native speakers and they all provided informed consent before starting the experiment. The study was approved by the local Psychology Ethic Committee. All methods were carried out in accordance with relevant guidelines and regulations. Data and additional information about the sample are available at https://osf.io/8u2yz/?view_only=da3d7536ed114101bbb76bb85f766856. The study was run in February 2022.

### Procedure

All participants were initially presented with the memory confusion task. The task was identical to the one employed in Study 1, with the only exception that for half of the participants four faces were covered by a face mask, whereas for the other half of the participants four faces were covered by a large spot of about the same size and color of the masks employed in the other condition (see Fig. 1, panel A, B, and C). The presence of either masks or spots was manipulated between participants in order to prevent possible carry-over effect. Afterwards, participants reported their political orientation and demographic information as in Study 1.

### Results

All the reported analyses were two-tailed and the alpha level was set at 0.05.

#### Memory confusion task

The data from 10 participants were excluded from the analyses because they provided random responses in the memory task (i.e., 3 or less correct answers) indicating that they did not pay adequate attention during the presentation phase. This left 94 participants in the mask condition and 96 in the spot condition. Scores based on errors were computed as in Study 1, namely by multiplying between-category errors by 3/4. A 2 (type of error: within- vs. between-category) × 2 (condition: mask vs. spot) analysis of variance was carried out with the first factor varying within participants and the second between participants. A main effect of the type of error emerged, *F*_(1, 188)_ = 4.09, *p* = 0.045, *η*^2^_*p*_ = 0.021. Indeed, within-category errors (*M* = 5.81, *SE* = 0.20) were significantly more likely than between-category errors (*M* = 5.35, *SE* = 0.17). Although the main effect of condition was not significant, *F*_(1, 188)_ = 3.71, *p* = 0.055, *η*^2^_*p*_ = 0.019, participants tended to make more errors in the spot (*M* = 5.86, *SE* = 0.20) rather than face mask condition (*M* = 5.303, *SE* = 0.21). The interaction effect was not significant, *F*_(1, 188)_ = 2.71, *p* = 0.101, *η*^2^_*p*_ = 0.014. However, given the specific hypothesis of the study, we separately explored within- and between-category errors in the two conditions. In the face mask condition, within-category errors were significantly more likely than between-category errors, *F*_(1, 93)_ = 6.53, *p* = 0.012, *η*^2^_*p*_ = 0.066, whereas no significant effect emerged in the spot condition, *F*_(1, 95)_ = 0.073, *p* = 0.788, *η*^2^_*p*_ = 0.001.

As in Study 1, because there was relevant interindividual variability in the overall memory performance, we also analyzed the proportion of within-category errors out of the overall number of errors. An independent sample t-test showed a significant effect of the condition, *t*_(187)_ = 2.09, *p* = 0.038. Indeed, the observed value was higher in the face mask condition (*M* = 0.47, *SD* = 0.15) than in the spot condition (*M* = 0.42, *SD* = 0.13). Corroborating previous analyses, the proportion of within-category errors was higher than chance in the face mask condition, *t*_(92)_ = 2.55, *p* = 0.012, but it was not different from chance in the spot condition, *t*_(95)_ = -0.239, *p* = 0.811.

As for correct responses, a 2 (speaker: with vs. without the mask/spot) × 2 (condition: mask vs. spot) analysis of variance was carried out, with the first factor varying within participants and the second between participants. The main effect of the first factor (i.e., whether the speaker had something on the face or not) was significant, *F*_(1, 188)_ = 10.37, *p* = 0.002, *η*^2^_*p*_ = 0.052. Indeed, correct responses were more likely when nothing covered the lower part of the original speaker’s face (*M* = 5.80, *SE* = 0.17) as compared to when either a face mask or a spot was present (*M* = 5.25, *SE* = 0.20). There was also a significant main effect of the condition due to a better memory performance for participants in the mask (*M* = 5.88, *SE* = 0.23) rather than in the spot condition (*M* = 5.16, *SE* = 0.23), *F*_(1, 188)_ = 4.60, *p* = 0.033, *η*^2^_*p*_ = 0.024. However, no significant interaction effect between the speaker and condition factors emerged, *F*_(1, 188)_ = 1.61, *p* = 0.207, *η*^2^_*p*_ = 0.008, suggesting that both the mask and the spot impaired correct recognition to a similar extent.

As in Study 1, we computed a single score in relation to political orientation (α = 0.89; *M* = 30.70, *SD* = 19.82). Political orientation was not significantly correlated with the proportion of within-category errors in the memory confusion task, *r*_(189)_ = 0.11, *p* = 0.129.

## General discussion

The present findings demonstrate that people make use of the presence (vs. absence) of a protective face mask as a cue to encode information in memory. Indeed, verbal information provided by an individual appeared to be tagged as a function of whether the speaker was wearing or not a face mask, as indicated by the higher number of within-category errors in the memory confusion task^43^. This effect was evident both in Study 1 and in the face mask condition of Study 2. Findings from Study 1 are consistent with the idea that the issue of face mask use does not need to be made contextually salient in order to observe an impact on categorization processes. Findings from Study 2 also suggest that the observed pattern can hardly be explained in terms of the perceptual difference between faces with either a mask or not. When the lower part of the speaker’s face was occluded by a superimposed colored spot, there was no evidence that participants organized information in memory as a function of this manipulated factor^42^. In contrast, data are more consistent with the view that protective face masks are here spontaneously processed because they represent meaningful social objects. After the outbreak of the COVID-19 pandemic mask-wearing has become a normative behavior at both the injunctive (i.e., what should be done) and descriptive (i.e., what most people do) level^48^. Hence, conformity to or deviance from the norm can elicit moral emotions and judgments^37^. Previous work using a memory confusion paradigm has established that social categorization may be sensitive to behaviors that either violate or uphold moral norms^49,50^. People not only categorize others along well-explored group dimensions such as sex, age, or race, but they also cluster individuals as a function of their level of compliance with normative behaviors. In a similar way, adaptive categorization processes, as assessed in a memory confusion paradigm, arise in order to group other individuals as a function of their cooperative vs. competitive behaviors^47^, thus suggesting that similar dynamics may come into play when perceiving mask wearers and mask non-wearers.

Importantly, the observed social categorization appears to be a rather spontaneous process which is set in place even when the dimension is irrelevant for the task at hand. In turn, this spontaneous categorization can lead to relevant consequences as it has been shown that typical group biases (i.e., ingroup favoritism) emerge among both mask and non-mask wearers^51^, suggesting that the appraisal of individual behaviors in relation to the use of masks affects both cognitive and social processes.

The evaluation along all the personality traits that were here assessed confirmed that mask wearers were judged more positively as compared to targets without the face mask, confirming that the use of face mask is indeed a socially relevant behavior that strongly shapes evaluative judgments. Even though the differential evaluation of the two types of targets varied in strength as a function of the specific dimension (i.e., being lower in the case of sociability), the effects were generally rather strong and unrelated to participants’ political orientation. In sum, findings provide support to previous work attesting a bias in favor of mask wearers^14,24–28^, although it should be acknowledged that the sample recruited in the present study is not necessarily representative of the whole population and therefore conclusions should be taken with caution.

The findings from the present set of studies further point to the relevant impact of the presence of face masks on identity recognition^18,21^. The overall memory performance was indeed significantly impaired when masks partially covered the faces. However, this effect does not appear to be attributable to face masks as such. In contrast, it is more likely due to the reduced availability of facial information. Indeed, the presence of a meaningless spot on the lower part of the face led to a comparable decrease in recognition^16^.

Overall, findings provide further support and extend previous literature about the impact of face masks on trait inferences and on the identification of social targets. Most importantly, it is here shown that spontaneous categorization processes are triggered, grouping individuals as a function of their mask-related behaviors. The implementation of preventive behaviors has become an integral part of people’s daily lives, and much has already been done to highlight the possible effects on socio-cognitive processes^9,52–57^. Although there is a widespread hope that we could soon overcome the Covid-19 pandemic, there is still uncertainty about the future. New variants are emerging and predictive models warn about the likelihood of recurrent new pandemics^58,59^. It does become essential to continue to explore the multifaceted ways in which the adoption of preventive measures, such as face mask use, may affect social cognition.

## Data Availability

The datasets generated and analyzed during the current study are available in the OSF repository https://osf.io/8u2yz/?view_only=da3d7536ed114101bbb76bb85f766856.

## References

[1] Wang C, Horby PW, Hayden FG, Gao GF (2020). A novel coronavirus outbreak of global health concern. Lancet.

[2] Chan EY (2021). Moral foundations underlying behavioral compliance during the COVID-19 pandemic. Pers. Individ. Differ..

[3] Howard MC (2020). Understanding face mask use to prevent coronavirus and other illnesses: Development of a multidimensional face mask perceptions scale. Br. J. Health Psychol..

[4] Lu JG, Jin P, English AS (2021). Collectivism predicts mask use during COVID-19. PNAS.

[5] Pagliaro S (2021). Trust predicts COVID-19 prescribed and discretionary behavioral intentions in 23 countries. PLoS ONE.

[6] Pfattheicher S, Nockur L, Böhm R, Sassenrath C, Petersen MB (2020). The emotional path to action: Empathy promotes physical distancing and wearing of face masks during the COVID-19 pandemic. Psychol. Sci..

[7] Brooks SK (2020). The psychological impact of quarantine and how to reduce it: Rapid review of the evidence. Lancet.

[8] Saladino V, Algeri D, Auriemma V (2020). The psychological and social impact of COVID-19: New perspectives of well-being. Front. Psychol..

[9] Pavlova MA, Sokolov AA (2022). Reading covered faces. Cereb. Cortex..

[10] Van Bavel JJ (2020). Using social and behavioural science to support COVID-19 pandemic response. Nat. Hum. Behav..

[11] Carbon CC (2020). Wearing face masks strongly confuses counterparts in reading emotions. Front. Psychol..

[12] Carbon C-C, Held MJ, Schütz A (2022). Reading emotions in faces with and without masks is relatively independent of extended exposure and individual difference variables. Front. Psychol..

[13] Grundmann F, Epstude K, Scheibe S (2021). Face masks reduce emotion-recognition accuracy and perceived closeness. PLoS ONE.

[14] Marini M, Ansani A, Paglieri F, Caruana F, Viola M (2021). The impact of facemasks on emotion recognition, trust attribution and re-identification. Sci. Rep..

[15] Pazhoohi F, Forby L, Kingstone A (2021). Facial masks affect emotion recognition in the general population and individuals with autistic traits. PLoS ONE.

[16] Stephan BCM, Caine D (2007). What is in a view? The role of featural information in the recognition of unfamiliar faces across viewpoint transformation. Perception.

[17] Bennetts RJ, Johnson Humphrey P, Zielinska P, Bate S (2022). Face masks versus sunglasses: Limited effects of time and individual differences in the ability to judge facial identity and social traits. Cogn. Res. Princ. Implic..

[18] Carragher DJ, Hancock PJB (2020). Surgical face masks impair human face matching performance for familiar and unfamiliar faces. Cogn. Res. Princ. Implic..

[19] Fitousi D, Rotschild N, Pnini C, Azizi O (2021). Understanding the impact of face masks on the processing of facial identity, emotion, age, and gender. Front. Psychol..

[20] Freud E (2020). The COVID-19 pandemic masks the way people perceive faces. Sci. Rep..

[21] Noyes E, Davis JP, Petrov N, Gray KLH, Ritchie KL (2021). The effect of face masks and sunglasses on identity and expression recognition with super-recognizers and typical observers. R. Soc. Open Sci..

[22] Stajduhar A, Ganel T, Avidan G, Rosenbaum RS, Freud E (2022). Face masks disrupt holistic processing and face perception in school-age children. Cogn. Res. Princ. Implic..

[23] Thorley C, Acton B, Armstrong J, Ford S, Gundry M (2022). Are estimates of faces’ ages less accurate when they wear sunglasses or face masks and do these disguises make it harder to later recognise the faces when undisguised?. Cogn. Res. Princ. Implic..

[24] Christiani L, Clark CJ, Greene S, Hetherington MJ, Wager EM (2021). Masks and racial stereotypes in a pandemic: The case for surgical masks. J. Race Ethn. Politics..

[25] Guo K, Hare A, Liu CH (2022). Impact of face masks and viewers’ anxiety on ratings of first impressions from faces. Perception.

[26] Lau WK (2021). Face masks bolsters the characteristics from looking at a face even when facial expressions are impaired. Front. Psychol..

[27] Oldmeadow JA, Koch C (2021). Effects of face masks on person perception. Perception.

[28] Olivera-La Rosa A, Chuquichambi EG, Ingram GPD (2020). Keep your (social) distance: Pathogen concerns and social perception in the time of COVID-19. Pers. Individ. Differ..

[29] Biermann M (2021). Trustworthiness appraisals of faces wearing a surgical mask during the COVID-19 pandemic in Germany: An experimental study. PLoS ONE.

[30] Grundmann F, Epstude K, Scheibe S (2021). Face masks reduce emotion-recognition accuracy and perceived closeness. PLoS ONE.

[31] Malik S, Mihm B, Reichelt M (2021). The impact of face masks on interpersonal trust in times of COVID-19. Sci. Rep..

[32] Kemmelmeier M, Jami WA (2021). Mask wearing as cultural behavior: An investigation across 45 U.S. States during the COVID-19 pandemic. Front. Psychol..

[33] Mallinas SR, Maner JK, Ashby Plant E (2021). What factors underlie attitudes regarding protective mask use during the COVID-19 pandemic?. Pers. Individ. Differ..

[34] Carbon CC (2021). About the Acceptance of Wearing Face Masks in Times of a Pandemic. I-Perception..

[35] Dudarev V, Manaligod MGM, Enns JT, Todd RM (2021). In the hands of the beholder: Wearing a COVID-19 mask is associated with its attractiveness. Q. J. Exp. Psychol..

[36] Graso M, Chen FX, Reynolds T (2021). Moralization of COVID-19 health response: Asymmetry in tolerance for human costs. J. Exp. Soc. Psychol..

[37] Rosenfeld DL, Tomiyama AJ (2022). Moral judgments of COVID-19 social distancing violations: The roles of perceived harm and impurity. Soc. Psychol. Bull..

[38] Taylor S, Asmundson GJG (2021). Negative attitudes about facemasks during the COVID-19 pandemic: The dual importance of perceived ineffectiveness and psychological reactance. PLoS ONE.

[39] Gadarian SK, Goodman SW, Pepinsky TB (2021). Partisanship, health behavior, and policy attitudes in the early stages of the COVID-19 pandemic. PLoS ONE.

[40] Klauer KC, Wegener I (1998). Unraveling social categorization in the “who said what?” paradigm. J. Pers. Soc. Psychol..

[41] Sherman SJ, Castelli L, Hamilton DL (2002). The spontaneous use of a group typology as an organizing principle in memory. J. Pers. Soc. Psychol..

[42] Stangor C, Lynch L, Duan C, Glass B (1992). Categorization of individuals on the basis of multiple social features. J. Pers. Soc. Psychol..

[43] Taylor SE, Fiske ST, Etcoff NL, Ruderman AJ (1978). Categorical and con-textual bases of person memory and stereotyping. J. Pers. Soc. Psychol..

[44] Kerr J, Panagopoulos C, van der Linden S (2021). Political polarization on COVID-19 pandemic response in the United States. Pers. Individ. Differ..

[45] Simić A (2022). Bringing us closer together: The influence of national identity and political orientation on COVID-19-related behavioral intentions. Front. Psychol..

[46] Ma DS, Correll J, Wittenbrink B (2015). The Chicago face database: A free stimulus set of faces and norming data. Behav. Res. Methods..

[47] Pietraszewski D, Cosmides L, Tooby J (2014). The content of our cooperation, not the color of our skin: An alliance detection system regulates categorization by coalition and race, but not sex. PLoS ONE.

[48] Neville FG, Templeton A, Smith JR, Louis WR (2021). Social norms, social identities and the COVID-19 pandemic: Theory and recommendations. Soc. Personal. Psychol..

[49] van Leeuwen F, Park JH, Penton-Voak IS (2012). Another fundamental social category? Spontaneous categorization of people who uphold or violate moral norms. J. Exp. Soc. Psychol..

[50] Goyal N, Adams M, Cyr TG, Maass A, Miller JG (2020). Norm-based spontaneous categorization: Cultural norms shape meaning and memory. J. Pers. Soc. Psychol..

[51] Powdthavee N, Riyanto YE, Wong ECL, Yeo JXW, Chan QY (2021). When face masks signal social identity: Explaining the deep face-mask divide during the COVID-19 pandemic. PLoS ONE.

[52] Cartaud A, Quesque F, Coello Y (2020). Wearing a face mask against Covid-19 results in a reduction of social distancing. PLoS ONE.

[53] Dalmaso M, Zhang X, Galfano G, Castelli L (2021). Face masks do not alter gaze cueing of attention: Evidence from the COVID-19 pandemic. I-Perception.

[54] Dalmaso M, Castelli L, Galfano G (2021). Increased gaze cueing of attention during COVID-19 lockdown. iScience..

[55] D’Ascenzo S (2022). Does social distancing affect the processing of brand logos?. Brain Behav..

[56] Scerrati E, D’Ascenzo S, Nicoletti R, Villani C, Lugli L (2022). Assessing interpersonal proximity evaluation in the COVID-19 era: Evidence from the affective priming task. Front. Psychol..

[57] Villani C (2022). Wearing the face mask affects our social attention over space. Front. Psychol..

[58] Marani M, Katul GG, Pan WK, Parolari AJ (2021). Intensity and frequency of extreme novel epidemics. PNAS.

[59] Markov PV, Katzourakis A, Stilianakis NI (2022). Antigenic evolution will lead to new SARS-CoV-2 variants with unpredictable severity. Nat. Rev. Microbiol..

